# Fabrication and biological evaluation of three-dimensional (3D) Mg substituted bi-phasic calcium phosphate porous scaffolds for hard tissue engineering

**DOI:** 10.1039/d2ra04009c

**Published:** 2022-11-24

**Authors:** Munusamy Ramadas, Ravichandran Abimanyu, José M. F. Ferreira, Anbalagan M. Ballamurugan

**Affiliations:** Department of Nanoscience and Technology, Bharathiar University Coimbatore 641046 India balamurugan@buc.edu.in; Department of Materials and Ceramic Engineering, CICECO, University of Aveiro Aveiro Portugal

## Abstract

This work reports on the fabrication of three-dimensional (3D) magnesium substituted bi-phasic calcium phosphate (Mg–BCP) scaffolds by gel-casting, their structural and physico-chemical characterization, and on the assessment of their *in vitro* and *in vivo* performances. The crystalline phase assemblage, chemical functional groups and porous morphology features of the scaffolds were evaluated by X-ray diffraction (XRD), Fourier-transform infrared spectroscopy (FT-IR) and field emission scanning electron microscopy (FE-SEM), respectively. The sintered scaffolds revealed an interconnected porosity with pore sizes ranging from 4.3 to 7.28 μm. The scaffolds exhibited good biomineralization activity upon immersion in simulated body fluid (SBF), while an *in vitro* study using MG-63 cell line cultures confirmed their improved biocompatibility, cell proliferation and bioactivity. Bone grafting of 3D scaffolds was performed in non-load bearing bone defects surgically created in tibia of rabbits, used as animal model. Histological and radiological observations indicated the successful restoration of bone defects. The overall results confirmed the suitability of the scaffolds to be further tested as synthetic bone grafts in bone regeneration surgeries and in bone tissue engineering applications.

## Abstract 2

This work reports on the fabrication of three-dimensional (3D) magnesium substituted bi-phasic calcium phosphate (Mg–BCP) scaffolds by gel-casting, their structural and physico-chemical characterization, and on the assessment of their *in vitro* and *in vivo* performances.

## Graphical abstract

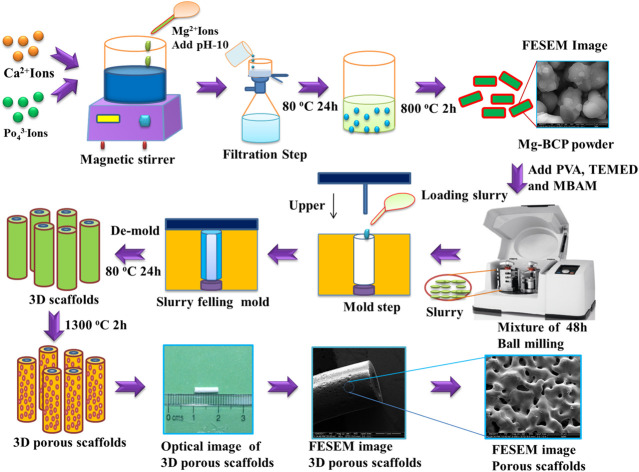

## 1. Introduction

Tissue engineering is an interdisciplinary emerging approach that tries to repair or replace bone tissue in locations where it has been damaged.^1,2^ Major orthopaedic issues such as aging-related bone defects or injuries, traffic accidents, fracture non-union, infections, and the removal of bone tumours have a negative impact on one's health and quality of life.^3^ Commercially accessible bone grafts are not universally applicable and a variety of issues need to be considered, including their eventual limited supply. The different bone graft options include autografts, allografts and synthetic grafts.^4^ Autografts are considered as the gold standard among the several grafting materials because of their osteogenic and osteoinductive growth factors, and a matrix that promotes bone adhesion.^5^ Being taken from the same person, the chances of infection are reduced. However, the longer surgery time required for harvesting, the morbidity at the donor location, and the limited amount of bone that can be transplanted are the main related shortcomings. The option for allografts (transplantable tissues coming from human donors) partially addresses the problems of autograft harvesting.^6^ However, this alternative comes frequently associated to risks of infection and high likelihood of host tissue non-union. Hence, a trending rise interest in the development of synthetic scaffolds for the regeneration of bone tissues has been witnessed in recent years.^7^

Bone tissue regeneration research has been heavily focused on developing innovative replacements for conventional bone grafts in order to overcome the limits of present therapeutic choices.^8^ Scaffolds created using a range of fabrication methods and biomaterials have been utilized to encourage and guide bone regeneration.^9^ It is still difficult to create a synthetic scaffold that fully resembles bone. Physiochemical qualities, bioactivity, biodegradability, osteoinductivity, and an interconnected porous structure that facilitates cell migration, proliferation and adhesion are all desirable characteristics in a synthetic scaffold.^10,11^ However, increasing porosity frequently compromises the mechanical quality that is crucial to preserving the structural integrity of the biomaterial.^12^

Three dimensional scaffolds for bone tissue engineering function have been developed using a variety of biomaterials; including stoichiometric hydroxyapatite (HAP, Ca/P ratio = 1.67) or beta tricalcium phosphate (β-TCP, Ca/P ratio = 1.50), and bi-phasic calcium phosphates (BCP) consisting of mixtures of HAP and β-TCP in different proportions. The use of scaffolds based on calcium phosphates (CaPs) is well justified considering that they make up the majority of the inorganic component of natural bones, with Ca/P ratios varying between 1.37 and 1.87.^13,14^ Due to their exceptional osteoconductivity, proliferation and biocompatibility, CaPs are also frequently utilized in simulated biomimetics.^15^ The main inorganic component of bone is not a chemically homogeneous substance as it also contains trace amounts of biologically relevant elements such as magnesium (Mg), strontium (Sr), zinc (Zn) and silicon (Si) that are crucial for bone growth, development and repair.^16–19^ The incorporation of these elements in bone graft substitute materials is expected to bring biological benefits for the engineered scaffolds. The replacement of calcium (Ca) by magnesium (Mg) in the apatite (HAP) lattice is likely to occur within a narrow molar composition range (up to around 10%).^20^ Heat treating the Mg–HAP at temperatures greater than 800 °C leads to crystalline phase changes with the formation of biphasic calcium phosphates (BCP)^21^. It is essential to find a way to fix the substation elements in the apatite (HAP) crystal lattices without (or with the least amount of) the formation of other less than biocompatible phases that contain magnesium (Mg) ions because the majority of apatite (HAP) devices are developed using high temperature treatments.^22^

The ultimate goal of tissue engineering is to develop three-dimensional (3D) scaffolds that can control the development of actual tissue structures and their distribution throughout the body in order to either replace or alter the behaviour of sick or damaged tissues.^23^ Scaffolds are produced by various conventional fabrication techniques such as gel casting, slip casting, 3D printing, manipulating ice crystallization, head welding and embossing were used.^24–28^ Recently, gel casting was one of the techniques employed for the development of 3D porous scaffolds. Gel casting process, slurries were prepared to a solid load containing three different organic monomer (binder, crosslinker and dispersant) solution to form castable slurry by ball-milling method. This technique has the advantage of allowed fully interconnected pores, pore size, good dimension accuracy and uniform distribution of porosity.^29,30^

In this work, we report on a gel casting method for consolidating three-dimensional (3D) Mg–BCP scaffolds with an interconnected pore network for hard tissue engineering. The chemical and physical properties of the scaffolds were assessed by different characterization techniques. The bioactivity and cytotoxicity of the scaffolds were evaluated *in vitro* using MG-63 cell line culture tests. The *in vivo* performance assessed by implanting the scaffolds in bone defects using a rabbits' model revealed their suitability for being used in clinical bone tissue engineering applications.

## 2. Materials and methods

### 2.1. Chemicals and reagents

Magnesium nitrate (Mg(NO_3_)_2_), calcium nitrate tetrahydrate (Ca(NO_3_)_2_·4H_2_O), diammonium hydrogen phosphate ((NH_4_)_2_HPO_4_) and ammonium hydroxide (NH_4_OH) reagents were supplied by S. D. Fine-Chemical Ltd, Mumbai-400030, India. Polyvinyl alcohol (PVA), *N*,*N*′-methylenebis-acrylamide (MBAM) and *N*,*N*,*N*′,*N*′-tetramethyl ethylenediamine (TEMED) were supplied by Loba Chemie Pvt. Ltd, Mumbai-400005, India. All chemical reagents were of analytical grade and were used as received without any further purification.

### 2.2. Synthesis of Mg–BCP

**Fig. 1:**
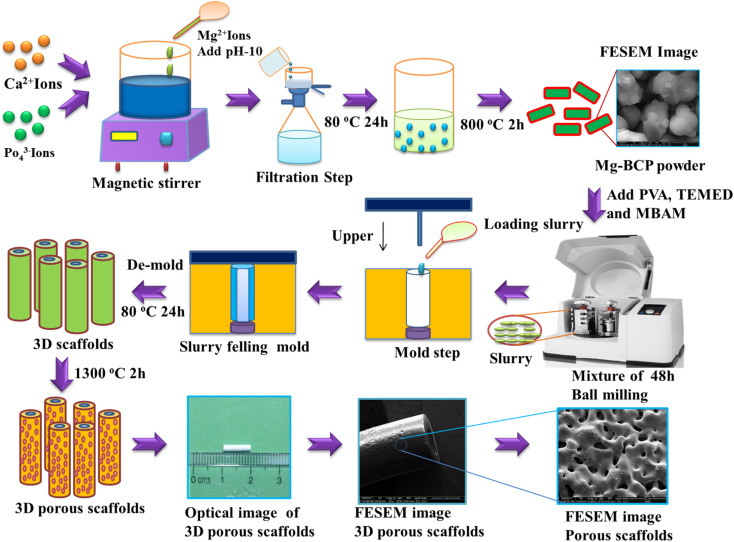
Schematics of the gel-casting process used to consolidate the Mg–BCP scaffolds, followed by sintering at 1300 °C for 2 h.

Undoped, and magnesium-substituted bi-phasic calcium phosphate (Mg–BCP) powders were prepared by the wet precipitation method schematized in Fig. 1. The (Ca + Mg)/P molar ratio in the precursor solution was set at (1.87 + 0.13)/1.3 = 1.538. Accordingly, a cationic solution was prepared by dissolving 1.87 mol of calcium nitrate tetrahydrate (Ca(NO_3_)_2_·4H_2_O) and 0.13 mol of magnesium nitrate (Mg(NO_3_)_2_) in 250 mL of double distilled water under magnetic stirring for 5 min. In parallel, the anionic solution was prepared by dissolving 1.3 mol of di-ammonium hydrogen phosphate (NH_4_)_2_(HPO_4_) in 100 mL of double distilled water. Then, the cationic solution was drop by drop added to the anionic one under vigorous magnetic stirring for 1 h, while keeping the pH at 10 by adding ammonium hydroxide (NH_4_OH) solution. After the stirring process was completed, the white precipitate was separated through a filtration step and then dried at 80 °C for 24 h in a hot air oven. The dried product was then calcined at 800 °C using a heating rate of 5 °C min^−1^, holding for 2 h at the maximum temperature, followed by natural cooling to room temperature (RT) inside the furnace.^31,32^ The calcined powder were finally ball milled and sieved using a planetary ball milling (Retsch) and a analytical sieve shaker (Retsch, AS 200), and stored in appropriate containers before being used for scaffolds fabrication.

### 2.3. Fabrication of undoped and Mg–BCP scaffolds

Three-dimensional (3D) undoped and Mg–BCP scaffolds were consolidated by gel-casting (Fig. 1). Slurries were prepared to a solid content of 35% for the gel by dispersing undoped or Mg–BCP powder in an aqueous solution containing 15% of organic monomers of polyvinyl alcohol (PVA)-binder, *N*,*N*,*N*′,*N*′ tetramethylethylenediamine (TEMED)-dispersant, and *N*,*N*′-methylenebisacrylamide (MBAM)-crosslinker at a molar ratio of 3 : 3 : 1 (PVA : TEMED : MBAM). To prevent agglomeration, the produced suspension was ball milled using Retsch planetary zirconium grinding jars at 350 rpm for 48 hours. Following the creation of a homogeneous suspension, a split-type (316L stainless steel) mould with dimension of 60 mm length and 30 mm diameter was cast using the material. The mould was then left to di-set at ambient conditions until the gelling process was complete. Gel was created in the mould by leaving it at room temperature. Following this procedure, the blocks were carefully de-moulded and placed in an environment that was ideal for conditioning in order to prevent cracking an uneven shrinkage of the 3D scaffold, which were 10 mm in length and 1.8 mm in thickness. The scaffold was dried at 80 °C of 24 hours before being sintered for 2 hours at 1100 °C and 1300 °C in a high temperature muffle furnace at a heating rate of 1 °C min^−1^.^33,34^

### 2.4. Characterization techniques

The XRD measurement were carried out with a Bruker AXSD-8 X-ray diffractometer with monochromatic CK, radiation (=1.5406) radiation source. Data were collected from two theta (degree) ranges of 20–60 at a scan rate 0.1 min^−1^ (degree per min). Fourier transform infrared FT-IR-Bruker 27, Germany instrument was employed to identify functional groups. The morphological characteristics of porous scaffolds were observed by using field emission scanning electron microscopy (FE-SEM) using (FEI Quanta-250 FEG) instrument with an accelerating voltage of 200 kV.

### 2.5. Bioactivity test in a simulated body fluid (SBF)

Bioactive were evaluated *in vitro* by immersing BCP and Mg–BCP scaffolds in simulated body fluids (SBF). The SBF stock solution was made by combining 142.0 mM NaCl, 4.2 mM NaHCO_3_, 5.0 mM KCl, 1.0 mM K_2_HPO_4_·3H_2_O, 1.5 mM MgCl_2_·6H_2_O, 2.5 mM CaCl_2_ and 0.5 mM Na_2_SO_4_ buffered at pH 7.4 with (CH_2_OH)_3_CNH_3_ and hydrochloric acid (HCl). Each BCP and Mg–BCP scaffolds, measuring 10 mm length and 1.8 mm in thickness diameter, was soaked in 45 mL SBF for 7 and 14 days at 37 °C. The specimens were withdrawn from the immersion tests, washed, and examined in a FE-SEM to see if there were any changes in surface morphology.^35^

### 2.6. *In vitro* study of BCP and Mg–BCP scaffolds

#### 2.6.1. Cell culture reagents

Dulbecco's modified Eagle's medium (DMEM), fetal bovine serum (FBS), penicillin (PEN) and phosphate buffered saline (PBS) were purchased from HiMedia Laboratories Pvt. Ltd. India. For MTT assay, 3-(4,5-dimethylthiazol-2-yl)-2,5-diphenyltetrazolium bromide were purchased from Bio Basic Canada.

### 2.7. Cell viability study on the scaffold

The cell line MG-63 was purchased from the National Centre for Cell Science (NCCS), Pune, India. Using the MTT assay, it was determined if the BCP and Mg–BCP scaffolds were cytotoxic to the Mg-63 cell lines, respectively. The Mg-63 cells were seeded in DMEM medium at the density of cells per well supplemented with 10% v/v FBS, 1% penicillin as a monolayer in grown at 37 °C under a humidified atmosphere of 95% air and 5% CO_2_. Cells were regularly passaged and maintained before including for the experiment. The MG-63 cells were seeded at a density of 1 × 10^4^ cells per well in 96 well plates and then treated with 100 μL of complete culture medium in the presence or absence of a series of increasing concentration 10, 30, 100, 300, and 1000 μg mL^−1^ of scaffolds for 24 h to test the cytotoxicity on the Mg-63 cells. A microplate absorbance spectrophotometer may be used to measure the coloured solution's absorbance at a specific wavelength of 570 nm.^36^

### 2.8. Implantation procedure

**Fig. 2:**
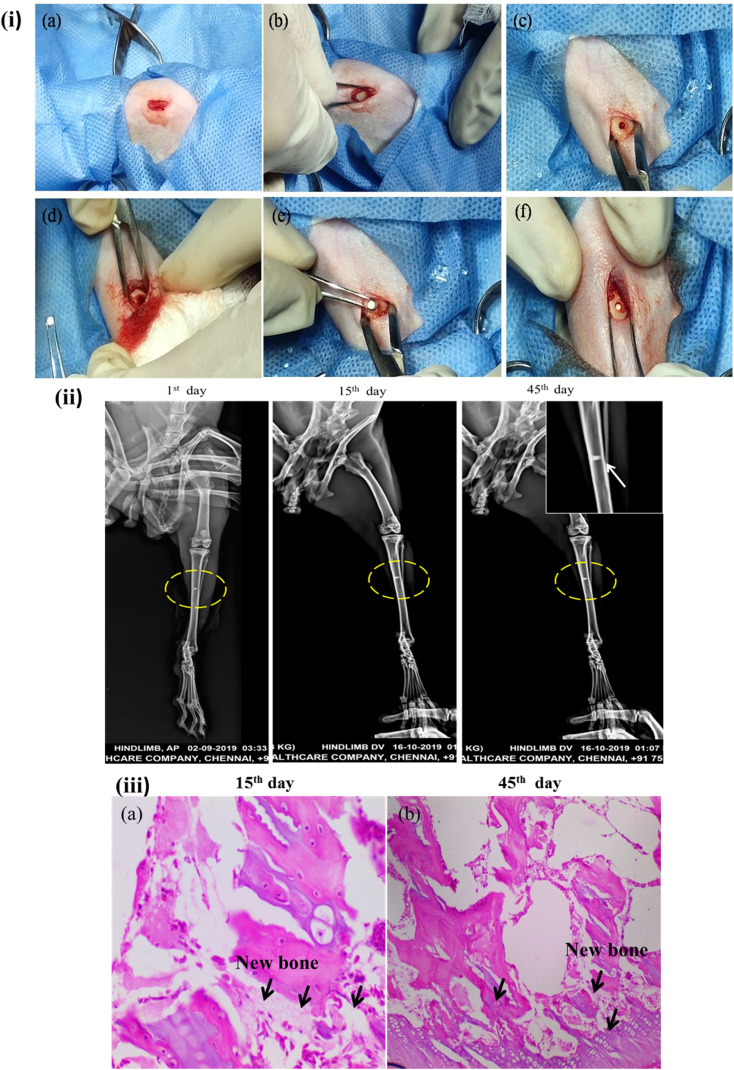
Bone repair ability of the scaffolds: (i) surgical implantation procedure of scaffolds into rabbits' tibia bone defects, (ii) X-ray radiographic images of the Mg–BCP samples at different post implantation of the points 1^st^ day, 15^th^ day and 45^th^ day. (iii) Histological analysis of implanted scaffolds 15^th^ day and 45^th^ day of surgery.

The three-dimensional porous Mg–BCP scaffolds with dimensions of 5 mm in length and 1.8 mm in thickness is created and fashioned to replace a bone. Scaffolds were sanitized before usage using gamma irradiation (Cancer Institute-WIA, Adyar, Chennai, Tamil Nadu, India). Two adult male New Zealand white rabbits (aged 20 weeks: body weight range: 2–2.8 kg) were used in the animal models. The rabbits were given regular food and water to eat. Prior to surgery, each rabbit had general anaesthesia with an intramuscular injection of 1 mL per kg 3% pentobarbital sodium. Surgery was maintained throughout the procedure. A blunt incision to separate the mussels was performed, revealing the tibia. A sterile stainless steel drill was used to make one hole in the tibia that was about 2 mm in diameter. The hole was then cleaned with physiological saline to get rid of any blood clots and bone fragments. The surgical process for implantation was carried out after the implant materials were inserted into the animals's hole (Fig. 2(i)). When the holes were filled, care was taken to prevent bleeding inside of them, and no material spilled into the surrounding area. Using chromic surgical catgut and silk thread, the hole was repaired by suturing the layer's muscles, subcutaneous tissues, and skin. The suture line was sealed with benzoin tincture. For the first 10 days following surgery, each animal got 0.5 mL of strep to penicillin. The animals were split into two groups: a control group that received no materials implanted, and a group that received the Mg–BCP scaffold. Under typical dietary settings, each animal was created for separately. Based on the following research, the effectiveness of the implanted materials was assessed.^37,38^

### 2.9. Conventional of radiological study

The density analysis of the implanted rabbits model was determined through X-ray screens radiographies (Kodak® Madrid, Spain) with Heliodent (70 kVp, 7 mA) was used at 30 cm fine focus receptor distance and anti-scatter grit with an exposure time of 0.30 s for molar region.^39^

### 2.10. Histological examination

The samples were stained with fluorescence for histological analysis after 45 days implanting. The samples were fixed in 4 percent formalin in 0.1 phosphate buffer with pH maintained at 7.4 for 12 hours. After 45 days of decalcification in 4 N formic acid, the specimens were imbedded in parafine, cut into 6 mm thick section, and stained with azan-mallory tri-chrome stain. The stained samples were examined using a fluorescent microscope.^40^

### 2.11. Statistical analysis

The data was analysed using one-way analysis of variance (ANOVA) (SPSS software) to identify the significance between different concentration samples. A P** < 0.05 were considered as statistically significance.

## 3. Results and discussion

### 3.1. X-ray diffraction analysis

**Fig. 3:**
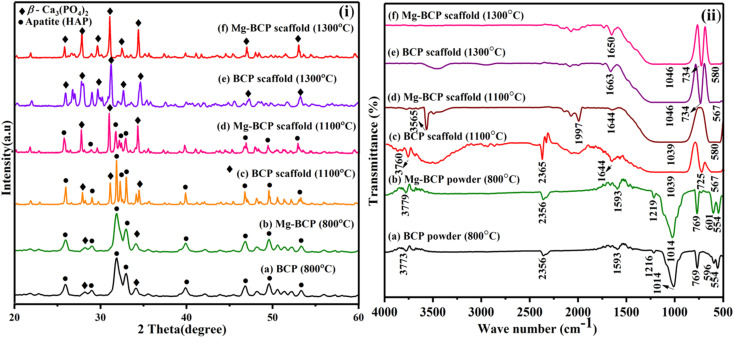
(i) XRD patterns and (ii) FT-IR spectra obtained for BCP and Mg–BCP scaffolds after heat treatment at 1100 °C and 1300 °C.

The X-ray diffraction analysis of as-synthesized BCP and Mg–BCP powders at 800 °C for 2 hours is shown in (Fig. 3(i)a and b). The main peaks seen in the spectrum are two theta (2*θ*) = 25.95, 28.22, 28.93, 31.90, 32.95, 34.09, 39.89, 46.82, 49.65, and 53.33° corresponds to (002), (214), (300), (210), (220), (310), (202), (213) and (018) which are in agreement with JCPDS no. 09-0432 for HAP and 09-0169 for β-TCP. The apatite (HAP) and β-tricalcium phosphate (β-TCP) mixture's crystal phase is well-crystalline, as seen by the sharp peaks BCP. For Mg–BCP, however, just the BCP peak was seen, indicating that the reduced Mg ion concentration means the Mg substitution in the BCP matrix does not appreciable change the crystal phase. This BCP and Mg–BCP powder were utilized to construct the three dimensional (3D) porous scaffold, and the finished 3D scaffolds received X-ray diffraction (XRD) analysis following heat treatment at 1100 °C and 1300 °C for 2 hours as indicated in (Fig. 3(i)c–f). The XRD patterns of the BCP and Mg–BCP scaffolds obtained at 1100 °C (Fig. 3(i)c and d) showed the primary refection indicative of the HAP and β-TCP combination of crystal structure. The porosity was not eradicated in the structure of the scaffolds that were sintered at 1100 °C, suggesting that the sintering was not processed. From 1300 °C onward, the sintering process began, and the pore size of the three dimensional (3D) scaffolds was reduced (Fig. 3(i)e and f). The presents of peaks at two theta (2*θ*) = 25.81, 27.86, 29.63, 31.11, 32.52, 34.36, 47.02 and 53.04° corresponds to (002), (214), (210), (211), (300), (220), (010) and (018) respectively. These findings suggest that the very porous scaffolds may be sintered and densified at 1300 °C without causing the production of any additional reaction products, such as bi-phasic calcium phosphate (BCP) crystal, besides apatite.^41^

### 3.2. FT-IR analysis

The FT-IR spectra of as-synthesised BCP and Mg–BCP powder in shown in (Fig. 3(ii)a and b). The presence of key functional groups such as phosphate (PO_4_^3−^), hydroxyl (OH^−^) and carbonate (CO_3_^2−^) in BCP and Mg–BCP powders are confirmed by FT-IR recorded at 800 °C for 2 hours. The bands at ∼554, ∼596, ∼769, ∼1014, ∼1216, ∼1593 and ∼2356 cm^−1^ are indicative of the existence of PO_4_^3−^ tetrahedral, whereas the OH – bands of apatite bands are discovered at ∼3773, ∼3779 and ∼3565 cm^−1^. These powders were used to make the fabrication scaffold, which was then submitted to FT-IR analysis after being heat treated for 2 hours at 1100 °C and 1300 °C (Fig. 3(ii)c–f). Similarly the FT-IR bands ∼567, ∼580, ∼725, ∼734, ∼1039, ∼1644, ∼1663, and ∼1650 cm^−1^ typical of PO_4_^3−^ tetrahedra of β-Ca_3_(PO_4_)_2_ is detected respectively at 1100 °C and 1300 °C. Moreover, the absence of OH^−^ groups distinctive of apatite phase is confirmed from the FT-IR patterns recorded at both 1100 °C and 1300 °C, thus confirming the transformation of apatite to Ca_3_(PO_4_)_2_. The yield of β-Ca_3_(PO_4_) from the scaffolds is evident from the characterization technique.^42^

### 3.3. FE-SEM analysis

**Fig. 4:**
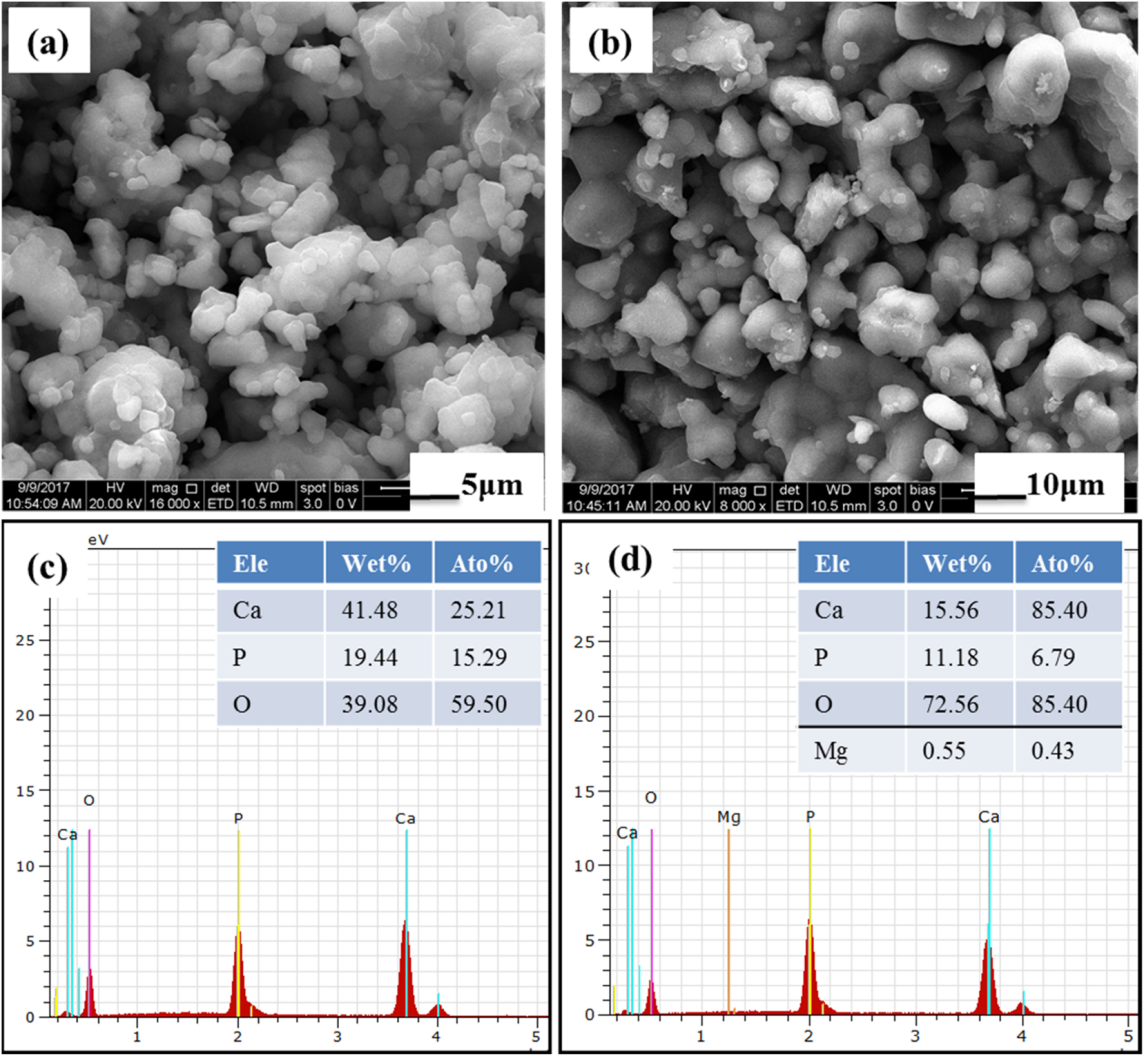
FE-SEM morphology of (a) BCP and (b) Mg–BCP powders: (c and d) EDS elements detected in the BCP and Mg–BCP respectively.

**Fig. 5:**
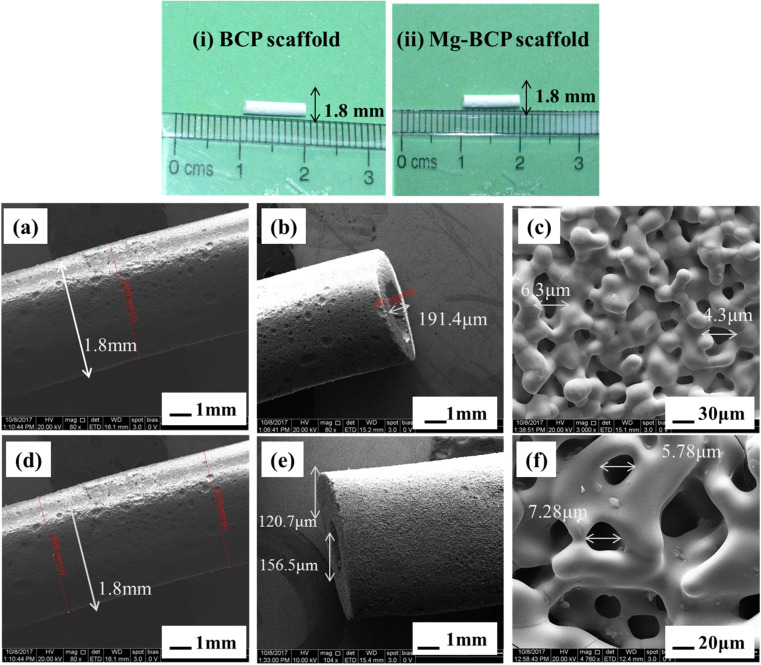
Photos of the (i) bi-phasic calcium phosphate (BCP) scaffold and (ii) magnesium substituted bi-phasic calcium phosphate (Mg–BCP) scaffolds: (a–c) pores BCP scaffold and (d–f) pores Mg–BCP scaffold from FE-SEM analysis.

The prepared BCP and Mg–BCP powders morphology analyses were carried out using FE-SEM. Fig. 4(a) and (b). Show in high magnification and low magnification of (a) BCP and (b) Mg–BCP powders are various shape and sizes. Undoped bi-phasic calcium phosphate (BCP) surfaces exhibit some aggregation and have a heterogeneous surface. However, particle size drops and agglomeration rises when Mg is introduced to the BCP matrix Fig. 4(b). As seen in Fig. 4(c) and (d), the EDS spectra clearly demonstrate the presence of carbon (C), oxygen (O), calcium (C), phosphate (P), and magnesium (Mg) elements. Scaffolds made of (i) BCP and (ii) Mg–BCP is shown in the photos (Fig. 5). After sintering at 1300 °C for two hours at a rate of 1 °C min^−1^, a three-dimensional (3D) scaffold with dimensions of 10 mm in length and 1.8 mm in thickness shape appropriate for a biological environment is created. Fig. 5(a–f) shows the porosity on the surface of the generated BCP and Mg–BCP scaffolds analysed by FE-SEM. The undoped BCP and Mg–BCP scaffolds include core pore that are 156.9–191.4 μm in diameter and outer pore that range in size 4.3–7.28 μm. The porous size of the undoped bi-phasic calcium phosphate (BCP) scaffold for hard tissue engineering should be regulated to facilitate proliferation and cell migrations. Additionally, compared to undoped BCP scaffold, the extremely porous Mg–BCP scaffold displayed improved biological characteristics and a higher mechanical strength. The replaced BCP scaffold displayed faster biodegradation and an increased Mg content, both of which point to potential use in hard tissue engineering.^43^

### 3.4. *In vitro* studies in synthetic physiological fluid

**Fig. 6:**
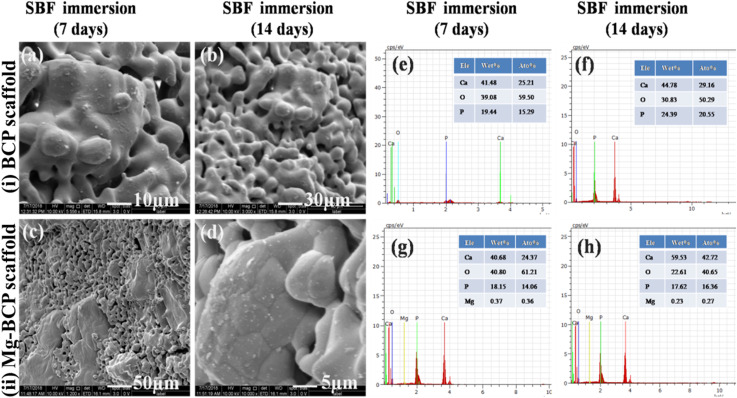
FE-SEM images of scaffolds made of (i) BCP (a and b), (ii) Mg–BCP (c and d), after immersion for 7^th^ (a–c) and 14^th^ days (b and d); (e–h) EDS elements detected in the BCP (e and f) and in the Mg–BCP (g and h) scaffolds after immersion in SBF for 7^th^ (e and g) and 14^th^ days (f and h).

*In vitro* bioactivity analysis of the fabricated scaffold was evaluated in simulated body fluid (SBF). The FE-SEM morphology of the BCP and Mg–BCP porous scaffolds after soaking in simulated body fluid (SBF) for 7 and 14 days are shown in (Fig. 6(a–d)). After 7 days of immersion, dissolution and precipitation were observed on the surface of the porous scaffold and it was confirmed that calcium deficient apatite. After the incubation period of 14 days, formation of apatite layer slightly increases and fully covered on the surface of the porous scaffold. The porosity of the scaffolds is a good sign of better bioactivity *in vitro*. The process of apatite layer formation is as follows: when a porous scaffold is immersed in SBF solution, rapid ion exchange occurs between the scaffold and physiological solution. During the immersion period, the cations (Ca^2+^) in the SBF solution are pulled by the anions OH^−^ and PO_4_^3−^ (hydroxyl and phosphate groups) on the surface of the porous scaffold. After this process, the surface of the scaffold gets positive charges and this positively charged ions on the surface again attracted negative ions (anions: OH^−^ and PO_4_^3−^) from the solution forms the dense apatite layer on the surface. The cations are easily getting attracted from the solution due to the more hydroxyl groups present in the surface of inorganic calcium phosphate porous scaffold. This repeated process leads to the formation of hydroxyl apatite layer on the surface of the porous scaffold. From the investigation, the results strongly suggest that the developed scaffold with interconnected pore (micro) could be used as vehicles and support for cell growth, proliferation and differentiation *in vitro* and also promising candidate material for bone tissue regeneration.^44,45^

### 3.5. MTT assay viability

**Fig. 7:**
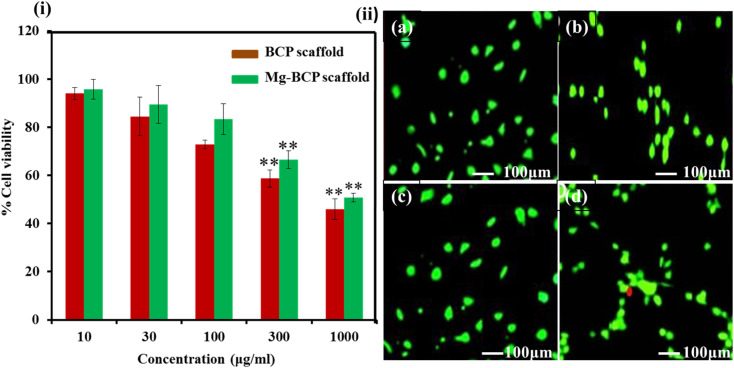
(i) *In vitro* cytotoxicity of undoped BCP and Mg–BCP scaffolds for different concentration (10 to 1000 μg mL^−1^) to MG-63 cells at 24 h. (ii) (a–d) Confocal microscopy images of live/dead analysis of at 24 h of culture.

The cell viability assays are fundamental steps in toxicology that elucidate the cellular response to a toxicant also provides information on the death of cells, metabolic activities, and survival of cells. MTT assay used to assess the cell proliferation and cytotoxicity of the as-prepared BCP and Mg–BCP scaffolds as shown in (Fig. 7(i)). MG-63 cell lines were exposed to BCP and Mg–BCP scaffolds at various concentrations of 10 to 1000 μg mL^−1^ for 24 h and the cytotoxicity was observed using MTT assays. The cell viability in MTT assay significantly reduced to pure 95 to 46% for the concentrations of 10 and 1000 μg mL^−1^, respectively. It can be demonstrated that the cell viability decreases gradually with the increase concentration of BCP and Mg–BCP scaffolds Fig. 7(ii). The absence of negative impacts in the BCP and Mg–BCP scaffolds is show by the prominent green fluorescence emission signal in all of the cell photographs. Dead cells produce red fluorescence whereas living cells emit the test circumstances. Despite being deadly, the Mg–BCP scaffold has no impact when used in other ways (Table 1).^46^

**Table 1:** Molar concentration of reactants: cationic and anionic ratio

Sample code	Cationic solution		Anionic solution	Ca/P molar ratio
	Ca	Mg	P	
Undoped BCP	2.0	0.0	1.3	1.538
Mg–BCP	1.87	0.13	1.3	1.538

### 3.6. Radiographic observation

After at the surgery, X-ray radiographs revealed callus had formed in tibia (hindlimb view) bone Mg–BCP scaffold in the defect region with uneven density and remnant materials, while in 1^st^ day, the bone defects remained obviously (Fig. 2(ii)). After 15^th^ day operation, bone formation was not observed in all samples. The margins of the fabricated porous Mg–BCP scaffolds were becoming unclear and some high-density signal emerged at the edge of the bone defect indicated that new bone was formed in the bone defect area in groups. At 45^th^ days after surgery, most of the implanted scaffolds had disappeared. Bone implanted materials have been replaced by new formed bone, which filled in the bone defects partially. Most of the regions in the bone defect area could not be observed in groups. In 45^th^ days, the boundary between the newly formed and host bone disappeared and complete healing of the bone defect was observed in all 2 rabbits groups. However, no bone formation was seen in up to 8 weeks after operation and only a small amount of bone formation was observed in the bone defect area at 45^th^ days groups (Fig. 2(ii)). Besides this, we also got X-ray score results. After 45 days, there were two samples above two points in group. For group Mg–BCP samples, it was 1^st^, 15^th^ and 45^th^ significant differences were observed when the three groups were compared in terms of bone formation. At the same time, Mg–BCP scaffolds presented a better result at different times when compared with ref. 47.

### 3.7. Evaluation of histopathology

The photomicrographs for histopathological analysis of the section of the Mg–BCP scaffolds implants are shown (Fig. 2(iii)). Elucidates the complete merging of the implant with the surrounding tissue the ossification was also observed. After the completion of the experiment's duration 15^th^ day and 45^th^ days the calcified histopathology section shows the formation of callus. Further, no significant difference was observed in the formation of new bone a temporary structure implanted Mg–BCP in the artificially created defects of rabbits left tibia of implantation section shows bony trabecula with expanded bone matrix (like callus). The histopathological observation indicated that after 45^th^ days of implantation, the bone tissue has completely filled the defect and a considerable amount of mineralized tissue was observed. The presence of osteoid tissue and active osteoblast along with the revised system indicates that the bone tissue apposition was almost complete and the osteoclastic activity indicated the bone remodeling process of newly formed bone.^48^

## 4. Summary and conclusions

In summary, three dimensional (3D) undoped BCP and Mg–BCP scaffolds were successfully fabricated using gel casting method. We have evaluated the porous microstructure, mechanical strength, bioactivity, osteogenesis and physiochemical properties of BCP and Mg–BCP scaffolds. The results indicated that Mg–BCP scaffolds with interconnected porous structure with a size ranging about 4.3–7.28 μm respectively. The *in vitro* analysis of the experiment showed that the fabricated three dimensional (3D) porous Mg–BCP scaffolds in a suitable proliferated and bioactivity of MG-63 cell lines and simulate body fluid (SBF). The results of this *in vivo* examination showed 3D porous Mg–BCP scaffolds implantation into the rabbit's tibia bone defect model. These results indicated that the developed three-dimensional (3D) Mg–BCP scaffold might be excellent options for bone tissue engineering.

## Conflicts of interest

There are authors declare no conflicts of interest.

## Supplementary Material
